# Osteogenesis Imperfecta Type II: The Lethal Newborn Form Diagnosed in the Postnatal Period

**DOI:** 10.7759/cureus.60945

**Published:** 2024-05-23

**Authors:** Hasnae Elhaddadi, Anass Ayyad, Sahar Messaoudi, Rim Amrani

**Affiliations:** 1 Department of Pediatrics, University Hospital Mohammed VI, Faculty of Medicine and Pharmacy, Mohammed First University, Oujda, MAR; 2 Department of Neonatology and Neonatal Resuscitation, University Hospital Mohammed VI, Faculty of Medicine and Pharmacy, Mohammed First University, Oujda, MAR

**Keywords:** newborn, fracture, ultrasound fetal, prenatal diagnosis, osteogenesis imperfecta

## Abstract

Osteogenesis imperfecta (OI) is a rare inherited skeletal disease, characterized by bone fragility and low bone density. There are several types of OI, varying in severity from benign to severe. We report a case of type II OI, which is a lethal form according to the Sillence classification. At birth, the newborn presented immediate respiratory distress. Postnatal examination and bone radiography confirmed the diagnosis of OI type IIA. The genetic analysis was done along with genetic counseling. Death occurred on day nine of life due to respiratory failure secondary to pulmonary hypoplasia.

## Introduction

Osteogenesis imperfecta (OI) is a group of connective tissue disorders characterized mainly by bone fragility. Its frequency is estimated at between 1/15,000 and 1/20,000 live births [1]. Several types of OI are generally distinguished, varying in severity from benign (type I) to lethal (type II). Types III and IV are severe but allow survival beyond the neonatal period, while types V to VII are characterized by a mild-to-moderate phenotype [2]. Diagnosis is based on the identification of pathogenic variants of alpha-1 type I collagen (*COL1A1*) and alpha-2 type I collagen (*COL1A2*) [3]. A multidisciplinary approach to care is recommended to treat not only fractures, reduced mobility, growth, and bone pain but also other extraskeletal manifestations [4]. We report the case of a newborn admitted to the neonatology and neonatal intensive care unit of the Mohammed VI University Hospital in Oujda for the management of respiratory distress with polymalformative syndrome.

## Case presentation

A female newborn was admitted to the neonatology and neonatal intensive care unit on the first day of his life with neonatal respiratory distress (Silverman score 3/10) and polymalformative syndrome. There was first-degree consanguinity between the parents. The pregnancy was poorly monitored (no morphological ultrasound) and delivered at term (depending on the date of the last menstrual period and the last ultrasound before delivery) by vaginal delivery, without suffering or neonatal infection. The mother and father were aged 38 and 42 years, respectively, and the mother was primiparous with no notable pathological history.

**Figure 1:**
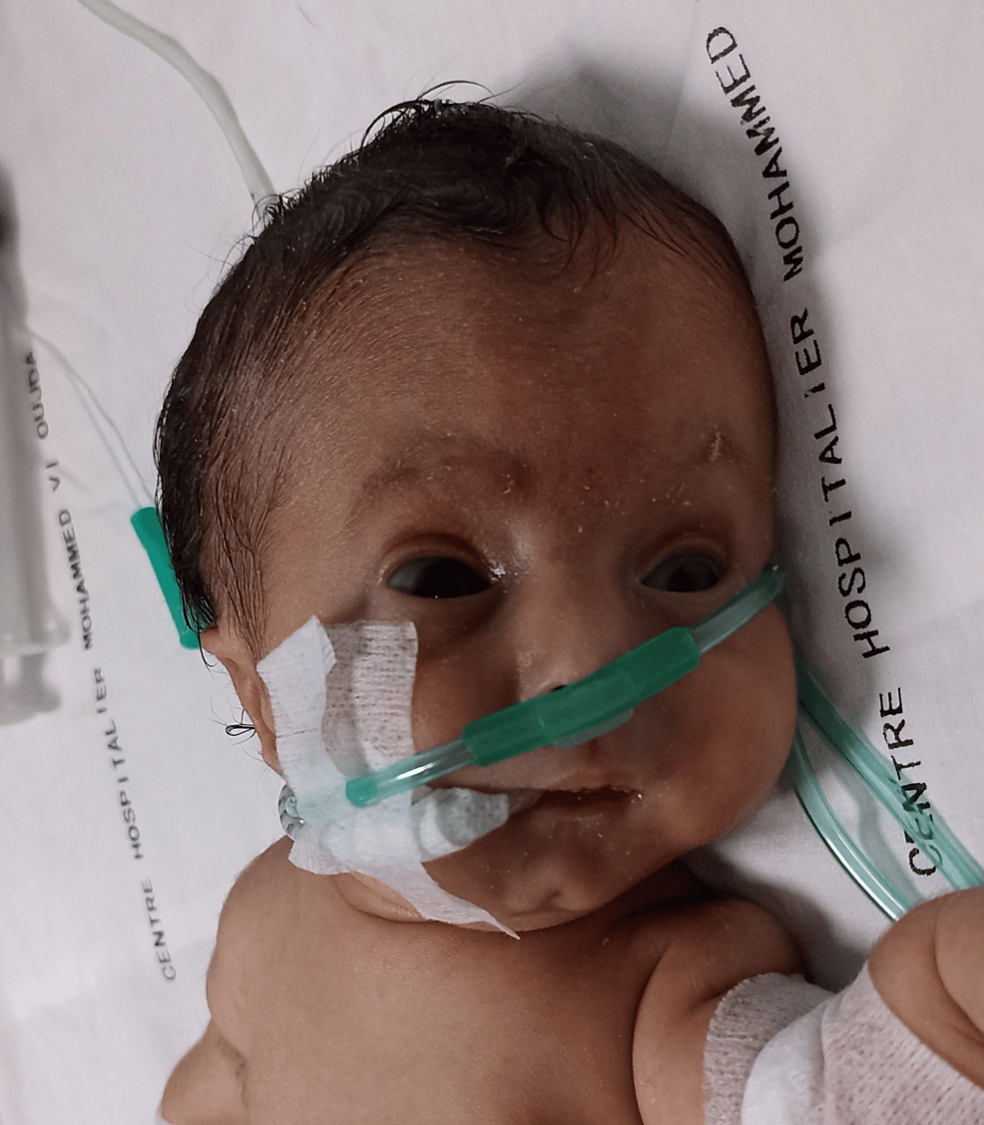
Blue sclerosis of both eyes, facial dysmorphia with retrognathism, triangular face, and prominent bridge of the nose.

**Figure 2:**
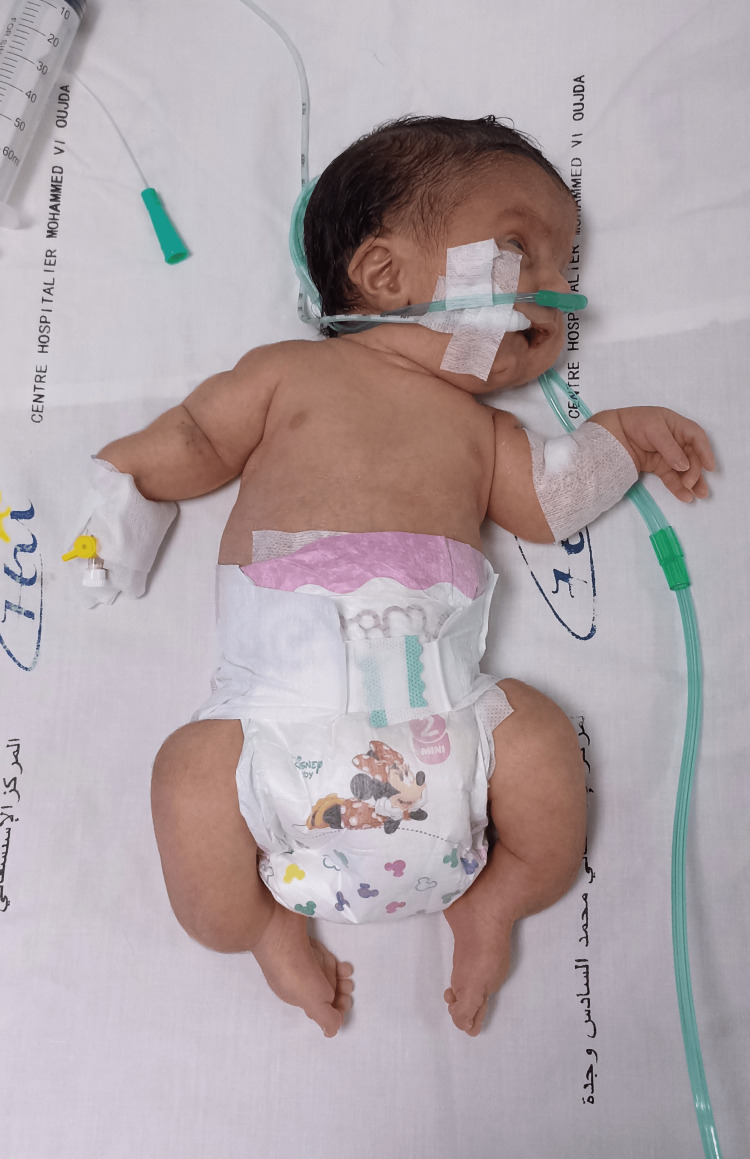
Short, deformed upper and lower limbs.

**Figure 3:**
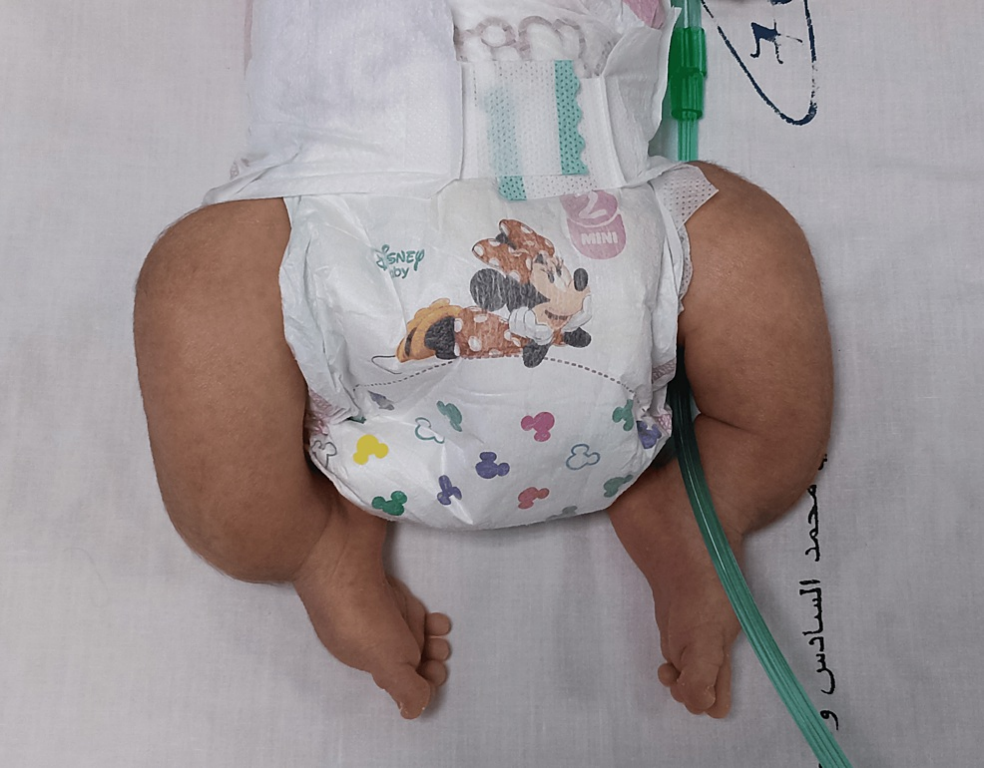
Deformities of both lower limbs in our patient: short, curved legs and wide thighs with angled femoral diaphysis.

On admission, clinical examination revealed a newborn with a weight of 2,500 g, polypneic at 45 cycles/minute, heart rate of 126 beats/minute, temperature of 37.6°C, capillary blood glucose of 1.24 g/L, and oxygen saturation of 92%. The newborn had a polymalformative syndrome (Figure 1) characterized by a facial dysmorphia consisting of a triangular face, retrognathism and a prominent bridge of the nose, transverse enlargement of the skull bones which were soft on palpation, a narrow bell-shaped thorax, short arms and legs, and an arcuate curvature of the lower limbs and wide thighs (Figures 2, 3).

Pleuropulmonary examination revealed no auscultatory rales. A cardiovascular examination revealed no murmurs or abnormal heart sounds. Ophthalmological examination revealed blue sclera in both eyes. No anorectal or finger abnormalities were noted. The rest of the somatic examinations were normal.

**Figure 4:**
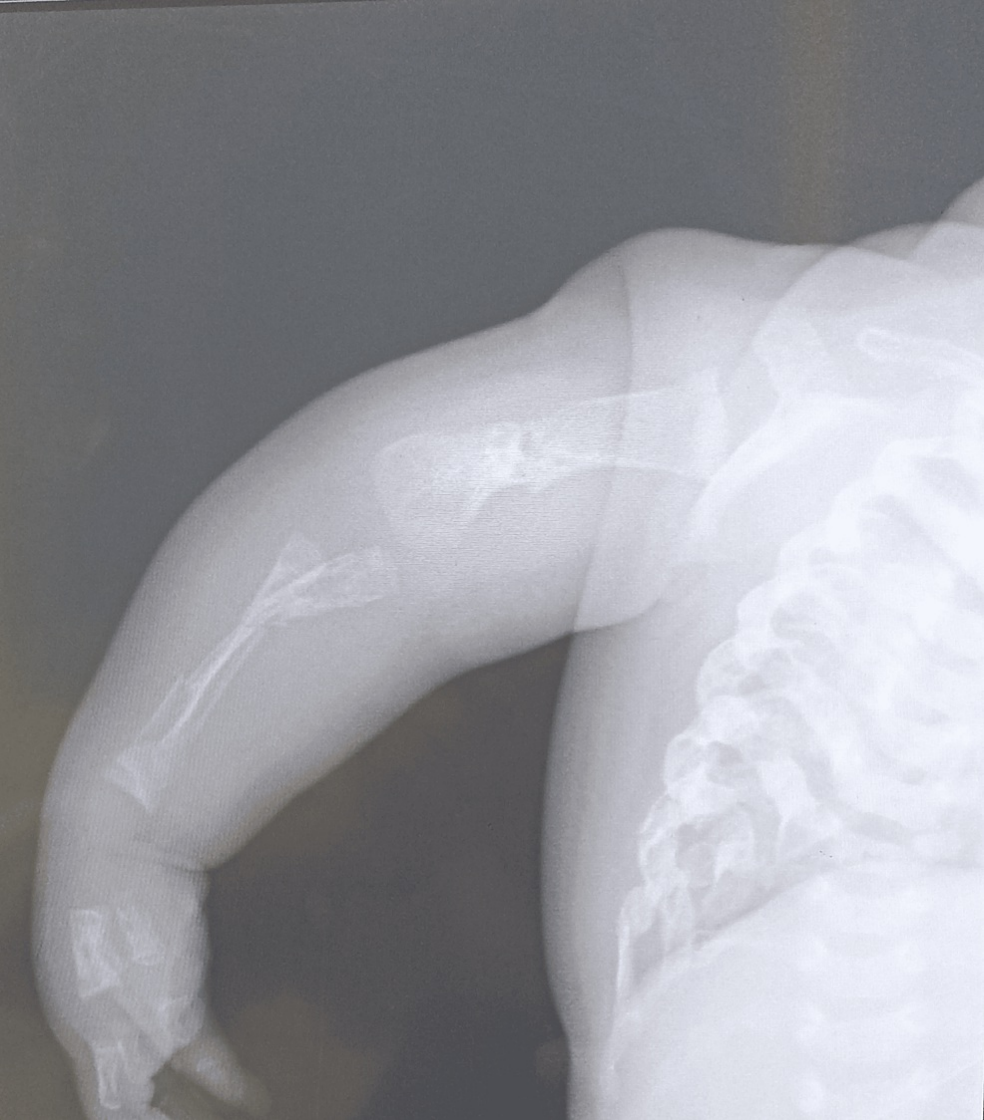
A narrow thorax, hypomineralized bone structure, pearly ribs with multiple rib fractures, and fractures and callus in the diaphysis of upper limb bones.

**Figure 5:**
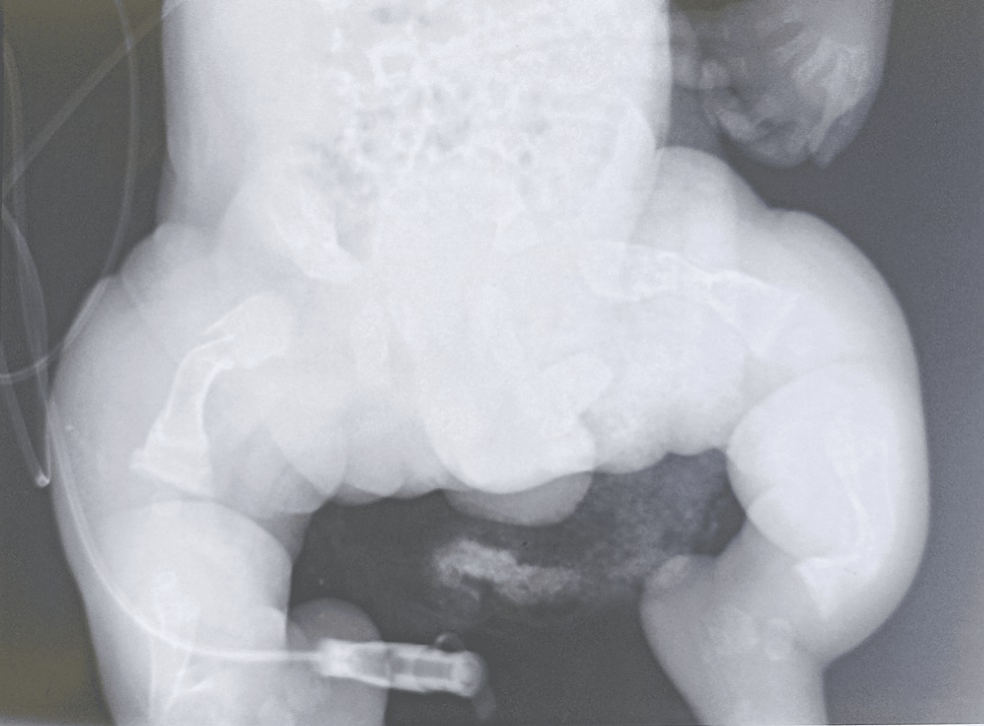
Fractures and malunion of both femoral diaphyses, micromelia with deformity, and curvature of the lower limbs.

Radiologically, standard skeletal X-rays revealed the following abnormalities: on the thorax (Figure 4): narrow thorax, hypomineralized bone structure, and pearly ribs with multiple rib fractures; on the limbs (Figure 5): bone deformities in the diaphysis of the lower limbs and bone demineralization with callus on the long bones (a sign of intrauterine fracture); in the skull: poor mineralization and worm-like bones. Transfontanellar ultrasound, transthoracic echocardiography, abdominopelvic ultrasound, and urinary tract ultrasound did not reveal any anomaly.

The blood count showed a white blood cell count of 8,570/µL, hemoglobin of 17.8 g/dL, platelets of 345,000/µL, and C-reactive protein of 10 mg/L. Liver function tests, hemostasis tests, renal function tests, and blood ionograms did not reveal any abnormalities (Table 1).

**Table 1:** Patient’s biological findings upon admission. CRP = C-reactive protein; SGOT = serum glutamic-oxaloacelic transminase; SGPT = serum glutamic-pyruvic transaminase; GGT = gamma-glutamyl transferase; ALP = alkaline phosphatase

Laboratory parameter	Values	Reference ranges
White blood cell (/μL)	8,570	4,000–10,000
Hemoglobin (g/dL)	17.8	14–18
Platelets (/μL)	345,000	150,000–450,000
CRP (mg/L)	10	<15
SGOT (IU/L)	12	5–35
SGPT (IU/L)	20	5–55
GGT (IU/L)	18	9–36
ALP (IU/L)	65	40–150
Sodium (mEq/L)	139	138–145
Potassium (mEq/L)	4,3	3.4–4.7
Calcium (mg/L)	91	88–108
Blood urea (g/L)	0,11	0.10–0.30
Blood creatinine (mg/L)	2,03	3.1–4.7
Prothrombin time (%)	89	70–100

Given the clinical and imaging findings, the diagnosis was that of OI in its lethal form (Sillence type IIA). A genetic analysis was used to confirm the diagnosis. The disease and its prognosis were explained to the parents, and genetic counseling was suggested. The evolution was marked by an aggravation of respiratory distress and death on the ninth day of life due to respiratory failure, secondary to pulmonary hypoplasia.

Informed consent was obtained from the patient’s family for the inclusion of images in this case report.

## Discussion

OI, also known as brittle bone disease, was described in 1835 by Lobstein and is an inherited disorder of bone formation. The clinical manifestations of OI include skeletal abnormalities such as fractures, osteopenia, bony deformities of the limbs, and articular laxity associated with variant extraskeletal abnormalities such as fragile blood vessels, blue sclerae, deafness, and dentinogenesis imperfecta [5].

The age of appearance and severity of the disease vary considerably from severe forms that are fatal during the perinatal period to benign moderate forms diagnosed much later in life. A classification of OI was proposed by Sillence and colleagues in 1981 according to disease progression and severity. Type II is the most severe form which leads to perinatal death [6]. In their classification of antenatal forms of OI, Maroteaux et al. divide lethal forms of OI into two groups: type L1 (typical lethal), corresponding to type IIA of Sillence classification, and type L2 (lethal with thin ribs), corresponding to type IIC of Sillence classification [7].

Prenatal diagnosis is essential in lethal forms of OI. It is primarily genotypic by analysis of fetal DNA. Samples are obtained either by amniocentesis at around 18 weeks’ gestation, or preferably by trophoblastic biopsy between 8 and 12 weeks’ gestation, enabling a more rapid diagnosis [7]. Antenatal ultrasound remains the examination of choice for detecting severe forms during a routine examination in the second trimester. It enables the diagnosis of type II OI according to the Sillence classification [8]. The antenatal diagnosis of a lethal form of type II OI is possible and evoked by the pathognomonic association of major micromelia of the four limbs with angulated diaphysis indicating multiple fractures and calluses. A very poorly ossified cranial vault, depressible to the pressure of the ultrasound probe, is responsible for too clear a visibility of the cerebral structures [8]. The visualization of a small, narrow thorax with chain-like ribs, fractured ribs, and even a callus is of prognostic value [7]. In such cases, fetal MRI can estimate lung volume, on which vital prognosis depends [9].

Perinatally, lethal forms of OI typically present with facial dysmorphia with micrognathism, a prominent bridge of the nose and blue sclerae, micromelia with deformity and curvature of the limbs, macrocephaly may be present, a defect in ossification of the skull which is soft to palpation, microcephaly is rarely present [7]. X-rays reveal generalized osteoporosis, confirming the shortness of the long bones and showing the presence of numerous calluses and fractures responsible for the deformities. The cranial bone is not mineralized, with occasional Wormian bones. The thin ribs, also the site of calluses, sometimes give a “bamboo-stem” appearance. The thorax is narrow and bell-shaped, with flattened vertebral bodies [10].

Genetic analysis is used to search for genetic mutations, which are often autosomal dominant and generally located in *COL1A1* or *COL1A2* (80-90%), which code for type I collagen chains, the main structural protein of bone [11]. Hereditary OI results in many cases from homozygous or heterozygous compound mutations in Two proteins, cartilage-associated protein (CRTAP) and leucine proline-enriched proteoglycan 1 (LEPRE1), which code for proteins involved in collagen biosynthesis. Genetic counseling can be difficult, as over 100 different mutations have been identified in patients with OI, and new mutations have recently been discovered [12].

The prognosis for type IIA is poor, with death in the perinatal period. Lethal OI is frequently associated with severe respiratory failure secondary to pulmonary hypoplasia [13], as observed in our case.

## Conclusions

OI in its lethal form (Sillence type 2) is a rare hereditary skeletal disorder. Its diagnosis is essentially antenatal and should be evoked on antenatal ultrasound in the presence of any bone anomaly or limb deformity. postnatal diagnosis is essentially clinically based on skeletal and extraskeletal manifestations and confirmed by genetic analysis looking for mutations. Genetic counseling should be offered in the event of a family history of OI.
